# Atrial scar on late gadolinium-enhanced imaging to predict electrical reconnection after pulmonary vein isolation for atrial fibrillation

**DOI:** 10.1186/1532-429X-18-S1-P201

**Published:** 2016-01-27

**Authors:** Hubert Cochet, Marjorie Salel, Stephanie Clement-Guinaudeau, Olivier Corneloup, Michel Montaudon, François Laurent

**Affiliations:** IHU Liryc - CHU / Université de Bordeaux, Pessac, France

## Background

Atrial fibrillation (AF) recurrences after pulmonary vein isolation (PVI) have been related to transient blocks leading to electrical reconnection. We assessed the accuracy of late gadolinium-enhanced (LGE) CMR in predicting, characterizing and localizing PV reconnection.

## Methods

LGE CMR was performed 3 months after PVI in 24 patients with paroxysmal AF (age 62 ± 9 years, 4 women). Imaging was performed 15 min after contrast in transaxial and sagital orientations using an inversion-recovery prepared and respiratory navigated 3D turboFLASH sequence with fat saturation (pixel size 1.25 × 1.25 × 2.5 mm). Scar burden (mL) was quantified on transaxial images using adaptive thresholding, with a threshold set 3SD above mean blood pool signal. In addition, scar circumferentiality was assessed visually by 2 observers in consensus, potential gaps being distributed on 4 sectors per vein (sup, ant, inf, post). 1 to 3 days after CMR, all patients underwent a systematic electrophysiological study, regardless of potential AF recurrence, in order to assess for PV reconnection. Each reconnection was characterized by high density activation mapping and distributed on the same sectors.

## Results

During the index procedure, complete isolation resistant to adenosine was successfully obtained on all 4 veins in all patients. At 3 months, PV reconnection was detected in 17/24(71%) patients, with a mean extent of 1.3 ± 1.0 veins and 1.8 ± 1.6 sectors per patient. Scar burden on CMR was 10.4 ± 4.5 mL (range 3.8 to 21.3), and scar gaps were present in 16/24(67%) patients. Global analysis demonstrated a strong relationship between scar burden and PV reconnection: using an optimal cut-off of 11 mL, scar burden predicted PV reconnection with a sensitivity/specificity of 82/100%, and an inverse relationship was found between scar burden and the extent of PV reconnection (R=-0.69, p < 0.001). In contrast, on regional analysis the relationship between scar gaps and PV reconnection was limited: on a vein-by-vein basis, scar gaps on CMR predicted PV reconnection with a sensitivity/specificity of 67/92%, and on a sector-by-sector basis the agreement between scar gaps on CMR and PV reconnection was only fair (k=0.47, p < 0.001).

## Conclusions

The assessment of scar circumferentiality around PV ostia on LGE CMR poorly predicts the presence and location of PV reconnections after PVI. However, the global quantification of scar burden relates to the presence and extent of PV reconnection. These results support the use of post-ablation CMR to identify patients likely to show AF recurrence, but additional research is desirable before considering guiding redo procedures from post-ablation CMR data.

**Figure 1:**
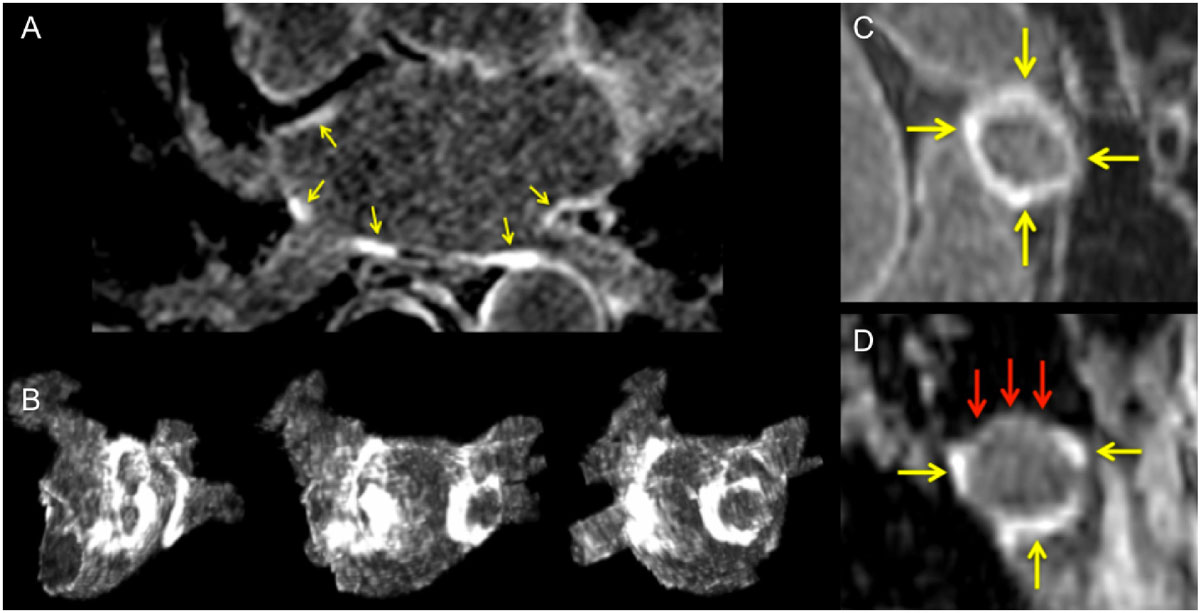
**LGE CMR acquired 3 months after pulmonary vein isolation for paroxysmal atrial fibrillation in a 65 year-old man**. Transaxial image (A) and maximum intensity projections (B) show scar on the ostium of each pulmonary vein. Scar burden was 15.3 mL. Multiplanar reformat on the ostium of right superior vein (C) shows circumferential scar with no gap (yellow arrows). Multiplanar reformat on right inferior vein (D) shows a scar gap on superior sector (red arrows). Systematic electrophysiological study performed 3 months after the PVI procedure (and 2 days after CMR) demonstrated no pulmonary vein reconnection.

